# GASTROINTESTINAL SCHWANNOMA: CASE REPORT

**DOI:** 10.1590/0102-6720201600030019

**Published:** 2016

**Authors:** Rafael Dienstmann Dutra VILA, Marlise Mello Cerato MICHAELSEN, Karine Sabrina BONAMIGO, Nilo Luiz CERATO, Valério Celso Madruga de GARCIA, Patrícia da Silva PASSOS, Adriano Calcagnotto GARCIA

**Affiliations:** Serviço de Coloproctologia, Hospital Ernesto Dornelles (Coloproctology Service, Hospital Ernesto Dornelles), Porto Alegre, RS, Brazil

**Keywords:** Gastrointestinal schwannoma, Mesenquimal tumor, Colorectal tumor

## INTRODUCTION

Schwannoma is a benign, neurogenic, slow-growing neoplasia, originated from Schwann cells, which are responsible by the myeline sheath on the peripheral nerves. This type of tumor is found more frequently on the central and peripheral nervous system and rarely occurs on the gastrointestinal tract^1^ ^,^ ^2^ ^,^ ^4^ ^,^ ^5^ ^,^ ^6^ ^,^ ^8^ ^,^ ^10^. Along with leiomyoma, leiomyossarcoma, gastrointestinal stromal tumor (GIST) and others, it makes part of the mesenchymal gastrointestinal tumors group^2^ ^,^ ^5^ ^,^ ^6^ ^,^ ^10^.

## CASE REPORT

Female, 74 years old, presented on the emergency room with a chief complaint of abdominal pain, nauseas, vomiting, prostration and dizziness with one week of evolution. She reported past history of diverticulitis and denied weight loss, hematoquezia or previous abdominal surgeries. During the physical examination, she complained of pain on deep upper abdominal palpation, although no abnormal mass could be detected. Proctologic examination and laboratory exams showed no abnormalities. 

**FIGURE 1:**
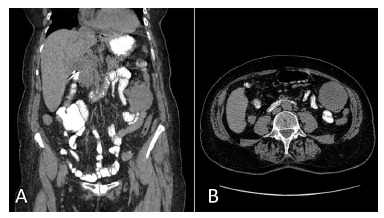
Splenic angle mass: A) CT coronal slice; B) TC axial slice (distal transverse colon)

Abdominal ultrassonography showed a nodular solid heterogenic type image on the left flank, with 6.1x5.6x4.3 cm. Investigation with contrasted computed tomography detected an delimited intramural lesion on the transverse colon, without invasion of surrounding organs (Figure 1).

Colonoscopy, with exploration until the cecum, showed not only sigmoidal diverticulus, but also an intraluminal bulging on the topography of distal transverse colon, with adjacent normal aspect mucosa, suggesting extrinsic mass growth and lumen compression. Since the research for metastatic lesions was negative, extended left colectomy was performed, with ressection of 18 cm intestinal segment, containing a 5.6x5.0x4.8 cm mass, located on the transverse colon, beside the splenic angle. The pacient had a satisfatory evolution, leaving hospital on the 6^th^ postoperative day. 

The anatomopathologic analysis have highlighted a fusiform cell mesenchymal lesion, extending from colon submucosa until its subserous layer, with moderate cell nucleus atypia and two mitosis per 50 high-power fields, without evidence of hemorrhage or necrosis.

Immunohistochemical research showed positive results for kit gene products (C-kit/CD117) and for glial fibrillary acidic protein (GFAP) and S-100 protein. The results for hematopoietic cells antigene (CD34), desmine and smooth muscle actin (CD117) were negative. This profile was compatible with a gastrointestinal tract schwannoma diagnosis.

## DISCUSSION

Schwannoma represents 0,2-1% of all gastrointestinal tract tumors, occuring more frequently on the stomach and rarely on colon and esophagus**^,^** ^1^ ^,^ ^2^ ^,^ ^4^ ^,^ ^5^ ^,^ ^6^ ^,^ ^9^ ^,^ ^10^. The mean age of incidence is around 50-60 years old, with equal gender prevalence^2^ ^,^ ^4^ ^,^ ^7^ ^,^ ^8^. It usually manifests itself by abdominal pain, constipation, gastrointestinal bleeding, weight loss, but sometimes it shows no significant syntoms^2^ ^,^ ^4^ ^,^ ^6^ ^,^ ^10^. It is classified as a mesenchymal gastrointestinal tumor^6^. 

The initial evaluation is made by computed tomography or nuclear magnetic resonance (NMR) to determine location, size, density of the lesion and attempt to identify metastasis^2^. Colonoscopy usually shows unharmed mucosa and an insert image sugesting extrinsic compression of intestinal lumen. However, all mesenchymal tumors have similar colonoscopic image aspect, making it difficult to set an specific diagnosis. In addition, a colonoscopy guided biopsy is not always able to collect sufficient amount of tissue to ensure a correct diagnosis^5^. Thus, anatomopathological and immunohistochemical research of the surgically resected lesion is mandatory^4^ ^,^ ^5^. 

**FIGURE 2:**
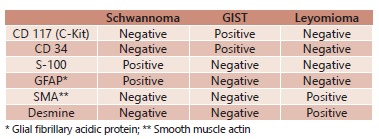
Mesenchymal tumor immunohistochemical profile

Therefore, in case of dealing with a resectable neoplasia, with high probability of mesenchymal tumor, surgical approach is indicated, with wide margin lesion resection, without necessity of lymphadenectomy, since the risk of metastasis in those cases is very low^1^ ^,^ ^2^ ^,^ ^4^ ^,^ ^5^ ^,^ ^6^ ^,^ ^9^ ^,^ ^10^. Considering the higher prevalence of GIST, the majority of schwannomas is misdiagnosed, until histological and immunohistochemical research and differentiation is concluded^4^ ^,^ ^5^. Schwannoma presents significant cell pleomorphism, lymphoid follicles, rare mitotic cells and rare necrotic spots. GIST shows high mitotic index, necrotic and hemorragic spots, without lymphoid follicles. Leiomyoma, on the other hand, does not show any of those characteristics^2^ ^,^ ^3^ ^,^ ^6^. The most important immunohistochemical markers are CD117, CD34, S-100 protein, GFAP, SMA and desmine. Schwannoma is S-100- and GFAP-positive, but CD117- and SMA-negative. GIST is CD117- and CD34-positive, S-100- and GFAP-negative. Leiomyoma is CD117-, CD34-, S-100- e GFAP-negative. However, the latest is desmine- and SMA-positive, which are negative markers on schwannoma and GIST^4^ ^,^ ^5^ ^,^ ^6^ ^,^ ^7^ (Figure 2).

The most important mesenchymal tumors prognosis factors, specially for GIST, are tumor size and mitosis index^2^. The less replicative and smaller is the neoplasia, the better is the prognosis. Radio and chemotherapy role in schwannoma remains uncertain; meanwhile, the recommended treatment is wide margin resection, without need of lymphadenectomy, mostly with excellent results ^5,9^.

## References

[1] Almeida MG, Hirschfeld APM, Farinha JCG, Roque MT, Ribeiro FLM, Mendonça PM, Volpiani JA (2005). Schwannoma de Reto Associado à Doença de Von Recklinghausen - Relato
de Caso. Rev bras Coloproct,.

[2] Friedman M, Nannegari V, Jones D, Valerian BT (2011). An Unusual Finding of Colonic Schwannoma. Practical Gastroenterology.

[3] Hou YY, Tan YS, Xu JF, Wang XN, Lu SH, Ji Y, Wang J, Zhu XZ (2006). Schwannoma of the gastrointestinal tract a clinicopathological,
immunohistochemical and ultrastructural study of 33 cases. Histopathology.

[4] Hsu WH, Wu IC, Chen Cy, Chiang SL, Chen HW, Wu DC (2009). Colon Schwannoma A Case. Report.

[5] Hung HY, Chiang JM, Chen JS, Tang R, Chen TS (2008). Schwannoma of the Colon: Report of Case and Review of the
Literature. J Soc Colon Rectal Surgeon (Taiwan).

[6] Kown MS, Seung SL, Ahn GH (2002). Schwannomas of the gastrointestinal tract clinicopathological features
of 12 cases including a case of esophageal tumor compared with those of
gastrointestinal stromal tumors and leiomyomas of the gastrointestinal
tract. Pathol Res Pract.

[7] Liegl B, Bennett MW, Fletcher CD (2008). Microcystic/reticular schwannoma a distinct variant with predilection
for visceral locations. Am J Surg Pathol.

[8] Miettinen M, Shekitka KM, Sobin LH (2001). Schwannomas in the colon and rectum a clinicopathologic and
immunohistochemical study of 20 cases. Am J Surg Pathol.

[9] Park KJ, Kim KH, Roh YH, Kim SH, Lee JH, Rha SH, Choi HJ (2011). Isolated primary schwannoma arising on the colon report of two cases
and review of the literature. J Korean Surg Soc.

[10] Xu M. (2011). Gastric Schwannoma: a rare Schwann cell tumour of the GI
tract. UWOMJ.

